# Regulating

**Published:** 1895-11

**Authors:** William J. Brady

**Affiliations:** Kansas City, Mo.


ARTICLE III.

### REGULATING.

BY WILLIAM J. BRADY, D. D. S., KANSAS CITY, MO.

Read before the Missouri State Dental Association at Pertle Springs, Missouri, July 15, 1895.

It is not the intention of this paper to be a treatise upon regulating, neither a mere display of cases treated; but I shall take the opportunity to urge upon you the closer study of this much neglected branch of dentistry, and try to show how much valuable work can easily be done by the ordinary practitioner, and hope that my effort may prove useful to some one. I am fully aware that regulating cases are but little desired by most practitioners; that this work is considered difficult and generally unsatisfactory, and that the financial return is considered very small in proportion to the time and labor required. But I believe the difficulties have been exaggerated; with correct appliances and judgment in their use, many simple cases can be treated with little expenditure of time and trouble, and made to pay as well as other kinds of work. The complicated cases should be referred, when possible, to a specialist in this branch, as this will always be found to the mutual advantage of all concerned. Regulating is one of the few things in dentistry that can be practiced as a specialty with satisfaction to all, and the profession should encourage the establishment of specialists by giving them hearty support; provided, of course, they are competent and attend strictly to their special work. The simple cases, however, will remain largely in the field of tha general practitioner, and he should know how to treat them.

I fully believe that the time will come when regulating will be as commonly practiced as crown- an bridge-work is to-day, and as successfully. If there were no better teaching in our schools on crown- and bridge-work than there is on regulating, it would be considered as difficult and unsatisfactory. Aside from the lack of knowledge on the subject, it has been difficult to secure proper apparatus for regulating until recently; but that trouble is removed now. There is no question but that the practical side of regulating has received its greatest growth since the introduction and development of Angle's system of regulation, and the placing upon the market of his appliances; thus putting proper apparatus within the reach of every dentist, as well as a complete system of treatment for all classes of cases, from the simplest to the most difficult. So complete is this system, so simple are the appliances, and yet so efficient, that it stands pre-eminently in advance of anything yet offered the profession in this line.

This paper will now deal with some of the simpler forms of irregularity, and their treatment according to this system.

It must be borne in mind that it is just as necessary to have a clear understanding of the case in a simple case as in a complex one. Here has been one of the great troubles in regulating. Dentists have attempted cases without thorough study, and have met failure before really beginning. As in anything else, first find out what is wrong before trying to fix it right. Study each malposed tooth and decide what movement is necesaary to bring it to its place; whether inward, outward, forward, backward, rotation, etc. It is thus possible to systematize the seemingly countless forms of irregularity into a few classes and instead of being all chaos and confusion, our cases assume regularity and our treatment becomes systematic.

Right here I want to enter my emphatic protest against the idea that each case is a case by itself and demands different treatment from every other case. The truth is that there is but little difference between cases where the same movement must be accomplished; and what is the proper thing in one case is also the proper thing in other cases like it. To accomplish the same movement requires about the same appliance, case after case, so when the movement required in the tooth or teeth is determined, the appliance suited to that movement is easily selected, and the great bugbear of regulating—the designing of appliances—disappears from view.

Of course there are other things to study than the mere movement required to bring a tooth to its normal position in the arch; it must be considered whether there is space enough to admit of the necessary movement; if not. how to get it: whether by extraction, or by expansion of the arch, and what occlusion would be secured by either method, and what influence it would have upon the fullness of the face, as by expansion of the arch a greater fullness would be obtained than by extraction, which sometimes is needed and sometimes is not wanted. The artistic effects possible upon the face by regulating are a great study in themselves.

The possible movements in regulating are as follows: Forward in the line of the arch, backward in line of the arch, from within (the arch) outward, from without inward, rotation, elongation and depression (in the socket.)

These movements may be single, as a tooth may be moved outward and rotated at the same time, or may be moved backward and inward and be elongated all at once, with possible rotation at the same time.

We will find almost everything we need for the performance of these movements in this lot of appliances, known as Angle's set No. 1.—*Western Dental Journal.*

